# Prevalence and risk factors of urinary incontinence among Estonian postmenopausal women

**DOI:** 10.1186/2193-1801-2-524

**Published:** 2013-10-17

**Authors:** Fred Kirss, Katrin Lang, Karolin Toompere, Piret Veerus

**Affiliations:** Tartu University Women’s Clinic, Tartu, Estonia; Department of Public Health, University of Tartu, Tartu, Estonia; Department of Epidemiology and Biostatistics, National Institute for Health Development, Tallinn, Hiiu, 42 11619 Estonia

**Keywords:** Urinary incontinence, Postmenopause, Risk factors, Prevalence

## Abstract

**Aims:**

To estimate the prevalence of urinary incontinence (UI) and to assess its risk factors among postmenopausal Estonian women.

**Methods:**

In 2004, 1363 women participating in the Estonian Postmenopausal Hormone Therapy Trial were asked at the closure visit to the trial physician about symptoms of UI. The type of incontinence was assessed with the help of a questionnaire, based on recommendations from the working group set up by the Finnish Gynaecological Association. Frequency characteristics were analysed by descriptive statistics. Risk factors were examined using logistic regression.

**Results:**

Mean age of study women was 53.3 years (min = 48, max = 67; SD 4.0). The prevalence of UI was 18.12% (95% CI: 16.07 - 20.17). Stress incontinence was diagnosed in 78.83% (95% CI: 73.32 - 84.33) and urge or mixed incontinence in 21.17% (95% CI: 15.67 - 26.68) of women who reported incontinence. Prevalence of UI slightly increased with age. Women who used hormone therapy (HT) (OR 1.67; 95% CI: 1.17 - 2.39), had had hysterectomy (1.73, 95% CI: 1.06 - 2.83), and those with secondary education (OR 1.87, 95% CI: 1.23 - 2.82) or basic education (OR 3.29, 95% CI: 1.80 - 6.02) had a higher risk for UI. Parity, having a BMI over 30 kg/m^2^, being a smoker or a former smoker, having diabetes and being physically or sexually active, tended to increase the risk of UI.

**Conclusions:**

About one in five postmenopausal women in Estonia reported to have UI. Risk factors linked with UI, its prevalence in other age groups and the impact of UI on quality of life deserve more research.

**Trial registration:**

Number: ISRCTN35338757

## Introduction

Urinary incontinence (UI) has been defined by the International Continence Society as: “the complaint of any involuntary leakage of urine” or as: “urine leakage seen during examination” (Abrams et al. 2002). Urogenital problems in the adult female population are common and have a significant impact on the physical, psychological and socio-economic aspects of life (Botlero et al. 2008). UI is one of the most significant urinary problems.

Depending its pathophysiologic process, UI is categorised into stress urinary incontinence (SUI), urge incontinence (sensory and motor) and mixed incontinence (Abrams et al. 2002). The differential diagnosis is important as it determines treatment and prognosis. A detailed understanding of the prevalence of UI in women is an essential step in reducing the huge impact of this condition.

The prevalence of urinary incontinence is estimated to be in the range of 12 - 69%, although estimates vary greatly in different countries and depend upon the age of the study group (Botlero et al. 2008;Andersson et al. 2004;Correia et al. 2009;Hunskaar et al. 2004;Irwin et al. 2006;Lasserre et al. 2009;Simeonova et al. 1999;Thom 1998;Cerruto et al. 2013). Various factors can cause pathophysiological changes in the muscular and fascial structures of the pelvic floor and lead to pelvic support defects and possible pelvic floor dysfunction. One of the main risk factors for developing urinary incontinence is believed to be pregnancy, childbirth and vaginal delivery resulting in injury to structures within the pelvic floor (Dolan & Hilton 2010;Serati et al. 2008). The relationship between trauma during vaginal delivery and incontinence, however, is not finally understood. Some population–based studies show an association between parity and urinary incontinence, others not.

The prevalence of SUI has been reported to increase at menopause and at menopausal age, and is more common in women than men, implicating menopause (Correia et al. 2009;Irwin et al. 2006;Iosif et al. 1981;Tinelli et al. 2010). In Estonia the average age at menopause is 50.3 years (Kirss et al. 2006).

Most epidemiological studies have shown clear association between urinary incontinence and obesity and certain medical comorbidities, particularly diabetes and stroke (Correia et al. 2009;Irwin et al. 2006;Dolan & Hilton 2010;Menezes et al. 2010). The association between incontinence and other factors/conditions is less clear. There is conflicting data about the potential roles of education, hysterectomy, constipation, and smoking in the development of incontinence (Correia et al. 2009;Irwin et al. 2006;Serati et al. 2008;Menezes et al. 2010;Rortveit et al. 2001;Waetjen et al. 2007).

The aim of this study was to estimate the prevalence (total and by type) and risk factors of UI in the Estonian postmenopausal female population. There are no earlier data available about the prevalence of urinary incontinence in Estonia.

## Methods

We used the data from Estonian Postmenopausal Hormone Therapy Trial. The trial was a long–term preventive trial of hormone therapy among healthy postmenopausal women during 1999–2004. Women were recruited into the trial by the means of a questionnaire which was mailed to 39713 postmenopausal women aged 50 to 64 and living in Tallinn (the capital of Estonia), Tartu, and in two counties surrounding these towns together with an information leaflet describing the trial and asking about their health status and willingness to participate in a postmenopausal hormone therapy trial. Potentially eligible 4295 women were randomised into four trial arms: 1) blind hormone therapy arm, 2) blind placebo arm, 3) non-blind hormone therapy arm, 4) non-treatment arm. Randomisation occurred before mailing the invitation to the recruitment visit in order to study the impact of blinding on recruitment. Randomised women were mailed an invitation to the recruitment visit, indicating whether they had been randomised to the blind or the non-blind sub-trial. As a result, altogether 1823 participants were recruited at three clinical centers between January 1999 and December 2001. Details of the trial design, randomisation, eligibility criteria, recruitment, and clinical follow-up of participants are described elsewhere (Veerus et al. 2006). Trial treatment was gradually stopped by May 2004, and closure visits took place from January 2004 to May 2004.

The mean duration of trial treatment was 3.6 years. Participants were followed by annual medical check-ups, annually mailed questionnaires and annual linkages to health registries. The trial design was approved by the Tallinn Medical Research Ethics Committee, Estonia and by the Ethics Committee of the University Clinic of Tampere, Finland. All participants signed written informed consent.

The main aims of the EPHT Trial were to study the effect of postmenopausal hormone therapy on the risk of cancer, cardiovascular diseases and bone fractures and the related use of health services, and quality of life. Approximately half of the trial women in the HT arms took more than 80% of allocated trial treatment during the last trial year. (Vorobjov et al. 2005) We analysed the prevalence of UI among postmenopausal women using the data from the closure visit of the EPHT Trial.

Participants of the current study were all 1363 women (74.8% of trial participants) who attended the closure visit to the trial doctor in 2004 when trial treatment was stopped. During the interview with the trial participants, the trial physicians filled in a questionnaire which included questions about health outcomes (whether the woman had been diagnosed with cancer, thrombosis, myocardial infarction, stroke, or diabetes), gynaecological operations, and a question asking if they had urinary leakage of any type. The 247 (18.12%) women who said that they had urinary leakage of any type had to answer ten more questions to specify the type of incontinence.

The questionnaire (Table 1) was based on recommendations from the working group set up by the Finnish Gynaecological Association (Mäkinen et al. 1992). If the score was less than or equal to 8, stress-incontinence was diagnosed. If the score was above 8, urge or mixed incontinence was diagnosed.

**Table 1:** **Female urinary incontinence score questionnaire**

Question		Score	
	0	1	2
1. Do you feel the urge to urinate before urinary incontinence occurs?	No	Slight	Strong
2. Does urinary incontinence occur in situations that don’t involve physical exercise?	No		Yes
3. Does urinary incontinence occur during physical exercise?	At once		In a moment
4. What is the amount of leakage urine?	Just drops	Little	A lot
5. Are you able to intentionally stop urinating?	Yes		No
6. Does pressure and moving around cause the urge to urinate?	No	Slight	Strong
7. Is there any urine left in the bladder?	No	Sometimes	Frequently
8. How many times do you urinate during the day?	5-7	8-10	11 or more
9. How many times do you urinate during the night?	0-1	2-3	4 or more
10. Have you suffered from urinary tract infection during the last two years?	No	1-2 times	Over 2 times

Quality of life was not assessed at the closure visit. Information about physical activity in the preceding 3 months, sexual intercourse in preceding 12 months, smoking, and other background information was obtained from the last annual questionnaire, sent by mail. The response rate to the final questionnaire was 81% (Veerus et al. 2008).

### Data analysis

Frequency characteristics were analysed by descriptive statistics. Risk factors were estimated by calculating crude and adjusted odds ratios (by means of logistic regression). A two-sided p value of 0.05 was considered to indicate statistical significance. Missing values in the UI questionnaire were imputed by multiple imputation using binary and ordered logistic regression. The Stata 9.2 software was used.

## Results

The mean age of women attending the closure visit and responding to the questionnaire was 53.3 years (min = 48, max = 67; SD 4.0). The main characteristics of the study group are presented in Table 2. About 40% of women were aged 62–66 years, forming the biggest age group, and women aged 57–61 years constituted one third of the respondents. Regarding place of residence, three quarters of women lived in the city. More than half of the respondents had secondary education, and over one third of women had higher education.

**Table 2:** **Main background characteristics of the study group, EPHT trial**

Variable	Number	%
**Age group, yrs**		
**52-56**	165	12.1
**57-61**	451	33.1
**62-66**	543	39.8
**67-69**	204	15.0
**Living place**		
**Town**	1017	74.6
**Country**	345	25.3
**Missing**	1	
**Education**		
**Higher**	458	33.6
**Secondary**	761	55.9
**Basic (8–9)**	143	10.5
**Missing**	1	
**Parity**		
**0**	129	9.8
**1**	322	24.5
**2**	648	49.4
**3 or more**	214	16.3
**Missing**	49	
**Smoking**		
**No**	981	72.1
**Yes**	182	13.4
**Previous**	197	14.5
**Missing**	3	
**BMI, kg/m** ^**2**^		
**<25**	484	30.1
**25-29**	562	41.9
**> = 30**	296	22.1
**Missing**	21	
**Diabetes**		
**No**	1069	96.9
**Yes**	34	3.1
**Do not know/missing**	260	
**Hysterectomy**		
**No**	1161	87.7
**Yes**	163	12.3
**Do not know/missing**	39	
**HT use**		
**Yes**	542	39.8
**No**	821	60.2
**Physical activity**		
**Yes**	360	26.4
**No**	890	65.3
**Missing**	113	
**Sexual intercourse**		
**Yes**	597	43.8
**No**	593	43.5
**Missing**	173	

*HT* hormone therapy.

The mean number of deliveries per woman was 1.8 (min = 0, max = 7; SD = 0.96). Almost half of women had had two deliveries and one fourth had had one delivery. The data revealed that 16.3% of women had had three or more deliveries and 9.8% of participants were nulliparous. A majority of participants (72.1%) were non-smokers, 13.4% were smokers and 14.5% were previous smokers. Around one fifth of the participants were obese with a body mass index (BMI) 30 kg/m^2^ or over and 41.9% of women had BMI of 25–29 kg/m^2^. About three percent of women were diabetic and around one in eight had had a hysterectomy. Nearly 40% of women were using HT, about a quarter reported to be physically active and about two in five having had intercourse within the preceding 12 months.

Data about the prevalence of urinary incontinence are presented in Table 3. The prevalence of urinary incontinence was 18.12% (95% CI: 16.07 - 20.17). The type of incontinence was estimated among 222 women of 247 who had reported UI. Among them, 150 had specified the type of incontinence. For the rest 97 women who had not answered some of the questionnaire questions, imputation was used to impute the missing values in the questionnaire (one answer was missing in 44 questionnaires, two in 20, three in seven, five in one, and for 25 respondents who did not fill in the questionnaire no imputation was used). After multiple imputation, the analysis showed stress incontinence in 78.83% (95% CI: 73.32 - 84.33) of the respondents and urge or mixed incontinence in 21.17% (95% CI: 15.67 - 26.68).

**Table 3:** **Prevalence and types of urinary incontinence in the study group**

	Number	Proportion	95% CI
**Incontinence**			
No	1101		
Yes	247	18.12	16.07 - 20.17
Missing	15		
**Type of incontinence**			
Stress	175*	78.83	73.32 - 84.33
Urge/mixed	47*	21.17	15.67 - 26.68
Missing	25		

*Calculated among subjects who had either completely or partly filled in the questionnaire (n = 222).

Table 4 presents the analysis of risk factors for UI. Risk of UI increased slightly with age but it was not statistically significant. Less educated women had a higher risk for UI. When women with basic education were compared with the ones with higher education, the adjusted risk for UI was about three times higher. HT use nearly doubled the risk of UI. Parity was also a risk factor, with women who had ever given birth tending to have a higher risk for UI when compared to women with no deliveries. Having a BMI over 30 kg/m^2^, being a smoker, being physically and sexually active tended to increase the risk of UI, although no statistically significant differences were found . A similar association, although with a higher risk estimate was seen for the association between diabetes and UI. Hysterectomy increased the probability of UI.

**Table 4:** **Association of different background factors with urinary incontinence**

Variable	Odds ratio (OR)			
	Crude	95% CI	Adjusted*	95% CI
**Age group, yrs**				
**52-56**	1		1	
**57-61**	1.27	0.77–2.10	1.29	0.68–2.46
**62–66**	1.45	0.89–2.37	1.69	0.89–3.20
**67–69**	1.69	0.97–2.95	1.72	0.83–3.56
**Education**				
**Higher**	1		1	
**Secondary**	1.39	1.01–1.91	1.87	1.23–2.82
**Basic (8–9)**	2.41	1.54–3.76	3.29	1.80–6.02
**Parity**				
**0**	1		1	
**1**	1.82	0.96–3.46	1.87	0.88–3.98
**2**	1.99	1.08–3.65	1.79	0.88–3.68
**3 or more**	2.33	1.20–4.53	1.72	0.77–3.83
**Smoking**				
**No**	1		1	
**Yes**	0.87	0.57–1.34	1.09	0.61–1.97
**Previous**	1.23	0.84–1.79	1.23	0.75–2.02
**BMI, kg/m** ^**2**^				
**<25**	1		1	
**25–29**	1.14	0.82–1.59	0.99	0.66–1.48
**> = 30**	1.83	1.27–2.63	1.05	0.65–1.72
**Diabetes**				
**No**	1		1	
**Yes**	1.96	0.89–4.32	2.24	0.94–5.32
**Hysterectomy**				
**No**	1		1	
**Yes**	1.60	1.09–2.35	1.73	1.06–2.83
**HT use**				
**No**	1		1	
**Yes**	1.17	0.88–1.54	1.67	1.17–2.39
**Physical activity**				
**No**	1		1	
**Yes**	0.98	0.71–1.35	1.09	0.74–1.60
**Sexual activity**				
**No**	1		1	
**Yes**	1.15	0.85–1.56	1.28	0.89–1.83

*adjusted for all other factors presented in the table.

## Discussion

The current study serves as the first step to quantify the disease burden caused by urinary incontinence in Estonia and to look into its risk factors. To our knowledge there have been no previous studies on prevalence and risk factors of UI among Estonian women. We found the prevalence of UI to be 18.12% among women aged 52–69 years. The prevalence of UI reported by us is significantly lower than rates reported in other studies among similar age groups (Botlero et al. 2008;Menezes et al. 2010;Waetjen et al. 2007). Comparing our results with population based studies, the prevalence of UI among women was remarkably lower than in France, Germany and UK (all around 40%), or in Spain and Portugal (23%) (Correia et al. 2009;Irwin et al. 2006). The EPIC study found a global prevalence of 18% in women aged above 17 years in Sweden, Italy, Canada, Germany and UK (Correia et al. 2009;Irwin et al. 2006). Swedish longitudinal data suggested that the prevalence of UI was approximately 20% and it did not differ significantly in women aged more than 19 years in 1991 and 2007 (Wennberg et al. 2009).

Our data among postmenopausal women at age 52–69 years show an even lower prevalence than among younger women in other counties. Only Andersson et al. in their study reported similar results to our study (19%) (Andersson et al. 2004). Partly, the UI prevalence in our study may be lower because the information was collected by a face-to-face interview in the physician’s office. Personal interviews are known to contribute to underreporting of sensitive issues, such as UI (Rhodes et al. 1995), and if it is not possible to carry out a clinical assessment of urinary dysfunctions, telephone interviews are preferred to mailed questionnaires to improve the completeness and accuracy of the data (Feveile et al. 2007). HT increased the risk of UI in our study, as also reported from the Nurses’ Health Study II study (Townsend et al. 2009) and Women’s Health Initiative trial (Hendrix et al. 2005). The prevalence of the UI in our study increased with age and was associated with low educational level, also shown in other studies (Hannestad et al. 2003;Danforth et al. 2006).

A significant association between parity and urinary incontinence has been reported earlier (Rortveit et al. 2001). Most women give birth in their twenties, but they complain with UI several years after delivery and prevalence of UI peaks in the middle-aged women (Danforth et al. 2006). An association is reported in the literature also between the number of the deliveries and UI (Hannestad et al. 2003). In our study we did not find that association to be significant, although the risk of UI was increased among women who had ever given birth.

In addition, such potentially modifiable lifestyle factors as obesity and smoking are associated with UI (Feveile et al. 2007). In our study, having a BMI over 30 kg/m^2^ and smoking tended to increase the risk of UI, although did not yield statistical difference, maybe because of a small number of patients. The risk of UI was also non-significantly higher among physically and sexually active women, a trend which could be explained by the fact that high-impact physical activities, whether recreational or occupational, can cause increased pressure on the bladder.

Hysterectomy is considered the starting point of pelvic floor symptoms, such as UI, constipation and sexual disturbances. Our study, similar to other retrospective and cross-sectional studies, reports a promotive effect of hysterectomy on UI. However, it is questionable whether there is a causal relationship because such an effect was not found in several prospective controlled studies (Gustafsson et al. 2006;Engh et al. 2006).

Diabetes mellitus, a metabolic disorder caused by an absolute or relative deficiency of insulin, is a debilitating and costly disease with multiple serious complications. A common complication of diabetes mellitus is diabetic bladder dysfunction which includes time dependent manifestations of storage and emptying problems (Daneshgari et al. 2009), it may also lead to UI. In our study we found a weak correlation, but the number of women with diabetes was very low and thus we can not make any conclusions.

There were a few limitations to our study. First of all, the study design that we used for this study was not suitable for establishing causality between risk factors and outcome. Secondly, there were some issues regarding representativeness of the study sample that need attention. Since Estonia has a relatively small population (1.3 million), the sample size in our study (n = 1363) was optimal. With regard to composition of the general Estonian female population of the respective age, the trial participants in our study were slightly more educated and urban than the general female population, and age groups 52–56 and 67–69 in the study were less representative of the general population. Also, the use of HT was higher among the trial participants than among the general population. These deviances from the general population composition, although statistically not significant, could have biased the results concerning prevalence of UI. For the analysis of risk factors for UI, adjustment was used to control for these deviances. Thirdly, the questionnaire used for the initial assessment of UI may not have been optimal. While concluding the trial in 2004, UI had not been studied in Estonia and there was no validated questionnaire in the Estonian language. Therefore, a questionnaire set up by the Finnish Gynaecological Association was adopted. In the future, a validated questionnaire would be preferable.

In addition to the limitations mentioned above, the study participants were all postmenopausal, which does not allow analysing the prevalence of UI in other age groups. Also, quality of life was measured only during the first trial years, but not at closure visit, which does not for an analysis of the effect of UI on womens’ life quality. As data about the mode of delivery among study participants was not available, it was not included in the analysis. Still, the impact of physical activity, sexual activity, and HT use on UI was analysed together with other background factors. Prevalence of UI was analysed among 1348 women, and the type of UI was calculated among 222 of women among those 247 who reported to have it. Imputation was used to substitute the missing data in the UI questionnaire for 97 women who reported to have UI, but did not respond to all survey questions. For the 25 women who had not responded to survey questions, no imputation could be used.

As a conclusion, there is a need in Estonia for a population-based longitudinal study to better ascertain the risk factors of urinary incontinence, quantify the burden of the problem in different age groups, as well as estimate the impact of UI on quality of life. The impact of modifiable risk factors for UI, e.g., smoking, BMI, hysterectomies and physical training, deserves additional research.

## Abbreviations

UI: Urinary incontinence
SUI: Stress urinary incontinence
BMI: Body mass index
HT: Hormone therapy.

## References

[CR1] Abrams P, Cardozo L, Fall M, Griffiths D, Rosier P, Ulmsten U, Van Kerrebroeck P, Victor A, Wein A, Standardisation Sub-committee of the International Continence Society (2002). The standardisation of terminology of lower urinary tract function: report from the standardisation Sub-committee of the international continence society. Neurourol Urodyn.

[CR2] Andersson G, Johansson JE, Garpenholt O, Nilsson K (2004). Urinary incontinence–prevalence, impact on daily living and desire for treatment: a population-based study. Scand J Urol Nephrol.

[CR3] Botlero R, Urquhart DM, Davis SR, Bell RJ (2008). Prevalence and incidence of urinary incontinence in women: review of the literature and investigation of methodological issues. Int J Urol.

[CR4] Cerruto MA, D’Elia C, Aloisi A, Fabrello M, Artibani W (2013). Prevalence, incidence and obstetric factors’ impact on female urinary incontinence in Europe: a systematic review. Urol Int.

[CR5] Correia S, Dinis P, Rolo F, Lunet N (2009). Prevalence, treatment and known risk factors of urinary incontinence and overactive bladder in the non-institutionalized Portuguese population. Int Urogynecol J Pelvic Floor Dysfunct.

[CR6] Daneshgari F, Liu G, Birder L, Hanna-Mitchell AT, Chacko S (2009). Diabetic bladder dysfunction: current translational knowledge. J Urol.

[CR7] Danforth KN, Townsend MK, Lifford K, Curhan GC, Resnick NM, Grodstein F (2006). Risk factors for urinary incontinence among middle-aged women. Am J Obstet Gynecol.

[CR8] Dolan LM, Hilton P (2010). Obstetric risk factors and pelvic floor dysfunction 20 years after first delivery. Int Urogynecol J Pelvic Floor Dysfunct.

[CR9] Engh MA, Otterlind L, Stjerndahl JH, Lofgren M (2006). Hysterectomy and incontinence: a study from the Swedish national register for gynecological surgery. Acta Obstet Gynecol Scand.

[CR10] Feveile H, Olsen O, Hogh A (2007). A randomized trial of mailed questionnaires versus telephone interviews: response patterns in a survey. BMC Med Res Methodol.

[CR11] Gustafsson C, Ekstrom A, Brismar S, Altman D (2006). Urinary incontinence after hysterectomy–three-year observational study. Urology.

[CR12] Hannestad YS, Rortveit G, Daltveit AK, Hunskaar S (2003). Are smoking and other lifestyle factors associated with female urinary incontinence? the Norwegian EPINCONT study. BJOG.

[CR13] Hendrix SL, Cochrane BB, Nygaard IE, Handa VL, Barnabei VM, Iglesia C (2005). Effects of estrogen with and without progestin on urinary incontinence. JAMA.

[CR14] Hunskaar S, Lose G, Sykes D, Voss S (2004). The prevalence of urinary incontinence in women in four European countries. BJU Int.

[CR15] Iosif S, Henriksson L, Ulmsten U (1981). The frequency of disorders of the lower urinary tract, urinary incontinence in particular, as evaluated by a questionnaire survey in a gynecological health control population. Acta Obstet Gynecol Scand.

[CR16] Irwin DE, Milsom I, Hunskaar S, Reilly K, Kopp Z, Herschorn S, Coyne K, Kelleher C, Hampel C, Artibani W, Abrams P (2006). Population-based survey of urinary incontinence, overactive bladder, and other lower urinary tract symptoms in five countries: results of the EPIC study. Eur Urol.

[CR17] Kirss F, Lang K, Tuimala R (2006). Feminine life-course of Estonian women born in 1937–47: a questionnaire survey. Acta Obstet Gynecol Scand.

[CR18] Lasserre A, Pelat C, Gueroult V, Hanslik T, Chartier-Kastler E, Blanchon T, Ciofu C, Montefiore ED, Alvarez FP, Bloch J (2009). Urinary incontinence in French women: prevalence, risk factors, and impact on quality of life. Eur Urol.

[CR19] Mäkinen J, Kujansuu E, Nilsson CG, Penttinen J, Korhonen M (1992). Virtsainkontinenssin arviointi ja hoito perusterveydenhuollossa (article in Finnish). Suom Laakaril.

[CR20] Menezes M, Pereira M, Hextall A (2010). Predictors of female urinary incontinence at midlife and beyond. Maturitas.

[CR21] Rhodes T, Girman CJ, Jacobsen SJ, Guess HA, Hanson KA, Oesterling JE, Lieber M (1995). Does the mode of questionnaire administration affect the reporting of urinary symptoms?. Urology.

[CR22] Rortveit G, Hannestad YS, Daltveit AK, Hunskaar S (2001). Age- and type-dependent effects of parity on urinary incontinence: the Norwegian EPINCONT study. Obstet Gynecol.

[CR23] Serati M, Salvatore S, Khullar V, Uccella S, Bertelli E, Ghezzi F, Bolis P (2008). Prospective study to assess risk factors for pelvic floor dysfunction after delivery. Acta Obstet Gynecol Scand.

[CR24] Simeonova Z, Milsom I, Kullendorff AM, Molander U, Bengtsson C (1999). The prevalence of urinary incontinence and its influence on the quality of life in women from an urban Swedish population. Acta Obstet Gynecol Scand.

[CR25] Thom D (1998). Variation in estimates of urinary incontinence prevalence in the community: effects of differences in definition, population characteristics, and study type. J Am Geriatr Soc.

[CR26] Tinelli A, Malvasi A, Rahimi S, Negro R, Vergara D, Martignago R, Pellegrino M, Cavallotti C (2010). Age-related pelvic floor modifications and prolapse risk factors in postmenopausal women. Menopause.

[CR27] Townsend MK, Curhan GC, Resnick NM, Grodstein F (2009). Postmenopausal hormone therapy and incident urinary incontinence in middle-aged women. Am J Obstet Gynecol.

[CR28] Veerus P, Hovi SL, Fischer K, Rahu M, Hakama M, Hemminki E (2006). Results from the Estonian postmenopausal hormone therapy trial [ISRCTN35338757]. Maturitas.

[CR29] Veerus P, Fischer K, Hovi SL, Karro H, Rahu M, Hemminki E (2008). Symptom reporting and quality of life in the Estonian postmenopausal hormone therapy trial. BMC Womens Health.

[CR30] Vorobjov S, Hovi SL, Veerus P, Pisarev H, Rahu M, Hemminki E (2005). Treatment adherence in the Estonian postmenopausal hormone therapy (EPHT) trial [ISRCTN35338757]. Maturitas.

[CR31] Waetjen LE, Liao S, Johnson WO, Sampselle CM, Sternfield B, Harlow SD, Gold EB (2007). Factors associated with prevalent and incident urinary incontinence in a cohort of midlife women: a longitudinal analysis of data: study of women’s health across the nation. Am J Epidemiol.

[CR32] Wennberg AL, Molander U, Fall M, Edlund C, Peeker R, Milsom I (2009). A longitudinal population-based survey of urinary incontinence, overactive bladder, and other lower urinary tract symptoms in women. Eur Urol.

